# Obtaining and Characterizing Bioplastic Films from Agro-Industrial Waste for Use in Manchego Cheese Packaging

**DOI:** 10.3390/polym18070838

**Published:** 2026-03-30

**Authors:** Maricela Villafaña-Jaramillo, Claudia Muro Urista, María Claudia Delgado Hernández, Rene Salgado-Delgado, Oscar F. Olea-Mejía

**Affiliations:** 1 Tecnológico Nacional de México/Instituto Tecnológico de Toluca, Avenida Tecnológico S/N Col Bellavista, Metepec 52140, Mexico; dd25282241@toluca.tecnm.mx (M.V.-J.); mdelgadoh@toluca.tecnm.mx (M.C.D.H.); 2 Tecnológico Nacional de México/Instituto Tecnológico de Zacatepec, Calzada Tecnológico 27, Centro, Zacatepec 62780, Mexico; rene.sd@zacatepec.tecnm.mx; 3 Centro Conjunto de Investigación en Química Sustentable, Universidad Autónoma del Estado de México, Carretera Toluca-Atlacomulco Km 14.5, San Cayetano, Toluca 50200, Mexico; ofoleam@uaemex.mx

**Keywords:** food industrial waste, starch, cellulose, avocado seeds, cornstalk, beet peels, carrot peels, bioplastic films

## Abstract

This research focuses on developing bioplastic films using agrifood industrial waste, which included starch from avocado seed, cellulose from cornstalk, carrot and beet peel, and pulp from a food company in México. The films were produced with a matrix of gelatin and glycerol, and different formulations of starch and cellulose. The films were characterized and tested as wrappers of Manchego cheese. The films containing starch are transparent; films with cellulose showed opacity and paper-like structure. Films containing starch–cornstalk cellulose showed the highest hydrophobic properties. In turn, films with carrot cellulose had the highest plastic properties with high elongation capacity and the lowest Young modules; films with starch and other celluloses showed the opposite data. The highest thermal capacity was observed in films containing cellulose from cornstalks and beet waste. In turn, the highest temperatures of transition, crystallization, and melting were registered in films containing starch. Films with starch and cellulose served well as wrappers of Manchego cheese, conserving 92% of the weight of cheese after 21 days of storage at 4 °C. All films were biodegradable in compost after 10 days, and they were degradable by physicochemical factors after 40 days.

## 1. Introduction

Bioplastic production has increased in recent years to replace conventional plastics, since they are produced from renewable sources, including agrifood waste and food industry waste from vegetables, seeds, and fruits, resulting in an important alternative for biocomposite production [1]. The waste is mainly composed of cellulose and starch extraction; however, it can also be integrated as biomass for biopolymer processing, providing materials for different applications [2]. Consequently, the production of bioplastics from waste positively impacts the environment and represents an alternative towards a circular economy [3].

Given the importance of using agrifood waste in bioplastic preparation, currently, there are several reviews describing its elaboration and properties [4,5]. Also, there are several investigations on bioplastics manufacture with different formulations of agrifood waste. Recent publications are highlighted: Orizano-Ponce et al. [6] used starch from Pouteria lucuma seeds to elaborate a bioplastic, whereas Lopes et al. [7] showed the use of carob and potato waste to obtain films for food packaging. Mroczkowska et al. [8] tested tomato peels and potato starch waste, producing a hydrophobic surface coating. Additionally, Ratna et al. [9] produced a bioplastic using waste of liquid tofu for food packaging. Hernando et al. [10] obtained bioplastics from oil palm trunk starch enhanced with citric acid-epoxidized palm oil oligomers. Zhang et al. [11] obtained cellulose from cornstalk to reinforce bioplastic composites for different uses.

Furthermore, previous studies from Hendrawati et al. [12] showed the use of rice husk to extract cellulose as a reinforcement in a cassava starch bioplastic containing PVA and ZnO. Ajayi et al. [13] used melon seed and peanut husk to obtain resistant materials. Maliha et al. [14] extracted sodium alginate from algae and combined it with microcrystalline cellulose from potato starch to obtain a bioplastic with improved water barrier permeation. Dorigato et al. [15] obtained a bioplastic from potato starch with a high elongation percentage. Choudhary et al. [16] elaborated edible bioplastic films with high elongation, using agarose and neem gum, hydroxyapatite, and sorbitol. Dewi et al. [17] improved the polymeric characteristics of chitosan films by adding cellulose fibers and starch.

Subsequently, Chowdhury et al. [18] used tamarind starch, blackberry seeds and ballas and licorice root to elaborate highly biodegradable bioplastics, whereas Guzman-Puyol et al. [19] used naringin and carbo methyl cellulose to obtain a bioplastic with UV blocking properties. Marichelvam et al. [20] used Prosopis Juliflora starch to produce a film with high tensile strength, whereas Bangar et al. [21], Martins et al. [22], and Araújo et al. [23] extracted starch from avocado seeds to measure their properties in film form.

In addition, reports of Shanmathy et al. [24] showed the use of taro starch reinforced with bentonite, enhancing the mechanical resistance of the material. Also, Chong et al. [25] reported the use of lignocellulosic fiber from corn waste to prepare and reinforce biocomposites with improved mechanical properties. Azmin et al. [26] tested cocoa pod husk cellulose incorporated with sugarcane bagasse fiber to enhance its mechanical properties. In turn, Sogut and Cakmak (2020) [27] used carrot fiber as a reinforcement in chitosan films, whereas Suffo et al. [28] found that the addition of beetroot waste on the polymer Carbocal^®^ increases rigidity and thermo-mechanical resistance.

Regarding bioplastic applications, Archundia et al. [2] tested biofilms of gelatin–chitosan containing guava leaf extract as meat packaging to extend its shelf-life. Lopes et al. [7] studied the use of a bioplastic from carob and potato waste for cheese and oatmeal cookies packaging. Products were preserved without significant changes after 5 days of storage. Chowdhury et al. [18] elaborated an antimicrobial film from starch from tamarind seed, berry and licorice root, showing positive effects against *E. coli*. Jridi et al. [29] analyzed the microbial effect of a film based in gelatin–pectin from orange peel to cover cheese during chilled storage.

According to the state of the art in biopolymer research, publications have grown exponentially; however, this information frequently refers to the integration of a single residue for developing such bioplastics. Furthermore, application-based results are often omitted, revealing a gap in this area and numerous opportunities for scientific contributions.

The present investigation has the objective of manufacturing bioplastics using various industrial wastes in the formulation (avocado seed starch and cellulose from cornstalk, carrot and beet peel) and to examine their application in food packaging.

This work addresses the need of repurpose agrifood waste from an industry localized in Mexico. This industry produces frozen, processed, and prepared foods, generating different types of waste, such as bagasse, bark, and non-selected components of vegetables, including carrots, beets, cornstalk and avocado seeds. Our goal is to expand current knowledge on bioplastics manufacture, incorporating multiple waste components and their application in food wrapping.

## 2. Materials and Methods

### 2.1. Materials and Reagents

Sodium hydroxide (NaOH) and sulfuric acid (H_2_SO_4_) were supplied by PQM Fermont S.A de C.V (Mirador No. 201. Col. Mirador. Monterrey, Mexico) (CAS No.1310-73-2 and CAS No. 7664-93-9). Sodium hypochlorite was supplied by Hycel (Av. Zoquipan No. 154, Col. Atemajac del Valle, Zapopan, Jalisco, Mexico) with 5% NaClO. Glycerol and gelatin were provided by Merck (Calle 5 No. 7, Fraccionamiento Industrial Alce Blanco. Naucalpan de Juárez, Estado de México, Mexico) (CAS No. 56-81-5 and CAS No. O9000-70-8).

### 2.2. Methods

#### 2.2.1. Waste Recollection and Reconditioning

In a single batch, raw food waste (10 kg in each case) was provided by a food industry localized in Lerma, Estado de México, Mëxico. The waste was immediately recollected after it was generated and was distributed in individual plastic bags of 1 kg containing only one vegetal, namely bagasse, peel, pulp and unselected components of carrot and beet, avocado seed, and cornstalk.

All waste was cut in small pieces and dried at 50 °C in a convection oven at approximately 1.5 m^3^/min of air-drying (Riossa HCF-62. Ecatepec, Estado de Mexico, México) for 24–36 h, ground and sieved, obtaining powder of each residue with a mean particle size of 160 μm. The materials were stored in airtight jars, for later use in the cellulose and starch extraction process.

#### 2.2.2. Starch Recovery from Avocado Seed

Starch extraction was carried out following the methodology of Martins et al. [22] with some modifications. The avocado seeds were washed and the shell covering the seed was removed. Avocado seeds were cut into small pieces. A total of 1 Kg of material was soaked with 1500 mL of distilled water for 12 h at 4 °C. Afterwards, the mixture was filtered and the solids were let to rest for 12 h. Using a siphon, the liquid was separated to retrieve the white sediment (starch). The starch was washed with deionized water at room temperature. After that, the material was dried at 40 °C for 24 h. The dry starch was ground and sieved using a 160-micron size sieve.

The starch was stored in glass recipients for later analysis and use. The proportion of starch was calculated in weight percentage, according to the weight of the sample waste (1 kg) and the weight of the obtained starch.

#### 2.2.3. Cellulose Recovery from Waste

The cellulose was extracted by alkaline and acid hydrolysis from each powder waste (carrot, beet, and cornstalk), using the modified methodology proposed by Raza et al. [30].

(1) Alkaline hydrolysis was applied, using a 2 wt% solution of sodium hydroxide in a 1:10 proportion referred to each type of residue. The mixture was stirred at 90 °C for 90 min. Subsequently, the mixture was filtered, obtaining a pulp, which was then washed with distilled water until the washing water reached neutral pH. (2). After, pulp from alkaline hydrolysis was put in another beaker for the acid hydrolysis application, using a 6 wt% solution of sulfuric acid in a 1:5 proportion at 90 °C for 60 min with agitation. The obtained product was identified as cellulose, which was filtered and washed with distilled water, until the washing water had the neutral pH. (3) Cellulose bleaching was applied. The cellulose was bleached using a 2.5 wt% solution of sodium hypochlorite in a 1:5 proportion and stirred at 90 °C for 15 min. The resultant pulp was dried at 40 °C for 12 h. After that, the dried material was ground and sieved using a 160-micron size sieve. The cellulose was stored in glass flasks for later analysis and use. The proportion of cellulose was calculated in weight percentage, according to the weight of sample waste (1 kg) and the weight of obtained cellulose.

#### 2.2.4. Bioplastics Preparation

**Figure 1:**
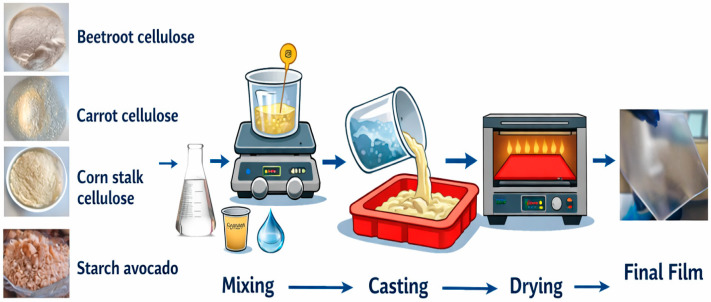
Elaboration of films based on avocado seed starch and cellulose from carrot, beet and cornstalk.

Figure 1 illustrates the procedure for producing the bioplastic films. Bioplastics were obtained, using as base water volume (250 mL), glycerol (1 mL) and gelatin (1.7 g) [31] and incorporating starch and cellulose with agitation at 90 °C and 120 rpm for 15 min (OHAUS 30541635 e-G52ST07C 120V AM Guardian™ 5000 Hotplate Stirrer. OHAUS Corporation, Parsippany, NJ, USA, EE. UU.) until a homogeneous mixture was obtained. The mixtures were placed in a silicone mold and dried in an incubator (17249 Heidolph high incubator 1000 unimax. Schwabach, Alemania) at 25 °C for 18 h. After that, the plastic film was removed from the mold and stored for later analysis and use.

Table 1 shows the formulation of 21 different films, using water, glycerol and gelatin as base material (film control A) and the incorporation of starch, cellulose or both from waste (films B−T), where B, C, D, and E films contain starch from avocado seeds; films F, G, H, and I contain cellulose from cornstalk; films J, K, L, and M are composites with cellulose from beet waste; films N, Ñ, O, and P contain cellulose from carrot waste; and films Q, R, S, and T are combined composites, containing starch and celluloses from waste.

#### 2.2.5. Determination of Physicochemical Properties of Bioplastic Films

Bioplastic films were characterized as explained below.

(A) Surface morphology was analyzed by scanning electron microscopy (SEM), using a JEOL microscope (JSM 6010 LV. Laboratory equipment supplier. Ciudad de México, México), operated at 10 Kv and 8.7 mm of working distance, without metal coating.

(B) Functional groups were identified by infrared spectroscopy (FTIR), using a FT/IR Jasco ATR Pro 4X. STEMART. Ciudad de México, México). The wave number range was 4000–400 cm^−1^ in ATR mode.

(C) Hydrophilicity was measured by contact angle test, using a goniometer by placing 1 µL of distilled water on the surface of the film (solid–liquid interface) at 18.5 °C. Photographs of the drops were taken in triplicate. The obtained image data was processed in Geo Gebra Image Editor to measure contact angle.

(D) Water solubility was measured by the gravimetric technique. The film was dried at 105 °C for 24 h, registering the initial weight (*Wi*). The dried samples were then immersed in deionized water 30 mL and stirred in a shaker incubator at 25 °C for 24 h. The films were weighted to report the final weight (*Wf*) [10]. The water solubility of the films was calculated using Equation (1), according to weight change. In turn, the swelling ratio was determined by immersing the dry film in deionized water (30 mL) at 25 °C for 1 h. The excess water of the films was drained with filter paper and the surface water was dried with absorbent paper. The films were weighted, registering the initial weight (*Wi*) and final weight (*Wf*); the swelling ratio of films was calculated using Equation (1), registering the weight change.

(1) $$Weight\;change\;\left(\%\right)=\frac{\left(Wi-Wf\right)}{\left(Wi\right)}\times 100$$

(E) Mechanical properties. Tensile strength, elongation at break, and Young modulus for films were measured in an INSTRON universal test machine, model Instron 3340 (825 University Ave, Norwood, MA, 02062-2643, USA), according to the ASTM D882-18 tensile standard for plastic films [32].

(F) Thermal properties. The films were thermally characterized via the thermogravimetric technique (TGA) and Differential Scanning Calorimetry (DSC), using a Thermogravimetric Analyzer TGA 550 Instrument TA. (EUAutomation, México) with ranges from 0 °C to 400 °C and an analyzer DSC 250 instrument (TA Instruments, New Castle, DE, USA) from 0 °C to 600 °C.

#### 2.2.6. Film Application in Cheese Wrapping

The films A–T were used as wrappers of sliced Manchego cheese to study their applicability. The test included the use of a commercial cheese wrapping (polyethylene, PE) for comparison.

One cheese slice was placed inside every film. The wrapped cheese samples were stored in a cold room (4 °C) and at room temperature (23–28 °C). Cheese preservation capacity of the films was evaluated by cheese weight changes, registering initial and final weight during 21 days of storage (Lopes et al. [7]) to obtain the kinetic behavior.

#### 2.2.7. Degradation of Bioplastic Films

Degradation of bioplastic films was measured by biodegradation (B) and ambient factor degradation, exposing the films to simultaneous effects, including water and solar light (W/S), water and air (W/A) and solar light and air (S/A), for 10 days [26].

The degradation was registered as weight change according to Equation (1).

The biodegradation of the films was studied for 28 days, analyzing the films every 7 days [18]. For the biodegradation tests, the films were cut into 0.02 m × 0.02 m sections, weighed, and placed under simulated composting conditions [20]. The compost was prepared with sawdust (40% by weight), rabbit droppings (30% by weight), mature compost (10% by weight), corn starch waste (10% by weight), sucrose (5% by weight), corn oil waste (4% by weight) and urea (1% by weight) [1]. In order to analyze the films, they were washed, dried, and weighed. Subsequently, weight loss was calculated using Equation (1).

#### 2.2.8. Statistical Analysis

The significance of the differences in the obtained values on three different film samples and three replicas of measurement was determined by analysis of variance (ANOVA) with Tukey’s test, at a 95% confidence level (*p* ≤ 0.05). The analysis was conducted using software MINITAB 22.1 (4 bit), 2024.

## 3. Results and Discussion

### 3.1. Biopolymers Films

**Figure 2:**
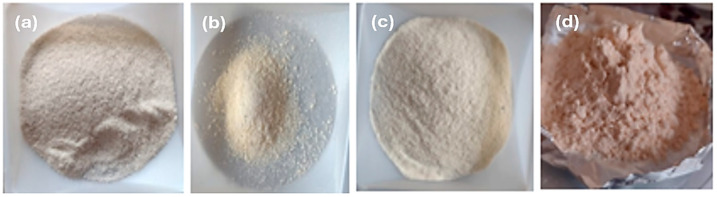
Images of raw material obtained from waste. (**a**) Beet cellulose, (**b**) carrot cellulose, (**c**) cornstalk cellulose and (**d**) avocado seed starch.

Figure 2 shows images of powdered cellulose from beet, carrot and cornstalk, showing a similar appearance. The image of starch from avocado seeds and celluloses are shown in Figure 1, displaying a finer powder of cellulose.

The cellulose yields from beet and carrot were analogous (24%, 25% respectively); however, cellulose from cornstalk exhibited the highest yield (52%). In turn, the yield of starch from avocado was 25%.

The yields obtained in the extraction of starch and cellulose were noteworthy, as they were recovered from food waste. The cellulose yields were different, since carrot and beet contain water and fiber, whereas cornstalk contains mainly fibers.

Previous studies of Sogut and Cakmak (2020) [27] and Reddy and Yang (2005) [33] reported similar cellulose yield (25%) from carrot and cornstalk; however, they used the whole carrot, and the alkali procedure was different. They used sodium hydroxide solution for 45 min at 95 °C and a ratio of 20:1.

Furthermore, there are studies showing higher yields of cellulose; however, the results are explained by the origin source. For example, Krishnan et al. [34], Latorre et al. [35], and El Khayat Driaa et al. [36] extracted cellulose from ixora coccinea, beetroot and walnut shells, indicating a 33.4% yield. However, they used physical pretreatment techniques by pulsed ultrasound with electric fields and high-voltage electrical discharges on the efficiency of alkali treatment, whereas Alemu et al. [37] studied cellulose nanocrystal extraction from teff straw, employing a two-stage alkali treatment followed by a bleaching process. In this case, the authors achieved a cellulose purity between 39.27% and 89.75%. In turn, Leite et al. [38] used a pretreatment (alkaline treatment–chelating treatment–bleaching), acid hydrolysis, and a final ultrasonic disintegration for cellulose extraction from cassava peelings, obtaining a 14.8% yield.

On the other hand, reports on starch extraction from avocado seeds indicated that the obtained yield in our research was analogous to the study of Martins et al. [22], obtaining 19.54% yield. However, other resources, such as cassava peelings, showed higher starch content (50.3%). Also, Leite et al. [38] revealed high starch yield (44.49% to 62.11%) from mango seeds, using the distilled water and alkali method, respectively [39]. Therefore, cellulose and starch yield depended on source origin, structure, and fiber content. However, the extraction method is important to increase the yields of these materials.

**Figure 3:**
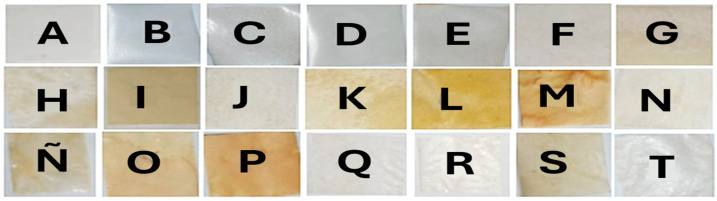
Images of 21 bioplastic films. According to Table 1, Film A is the film control, containing water, glycerol and gelatin. Film B–E are film A, containing different mass of starch. Film F–I are film A, containing different mass of cellulose from cornstalk. Films J–M are samples of film A, containing cellulose from beetroot. Films N–P are samples of film A, containing cellulose from carrot waste. Films Q–T are composites from film A, containing starch and cellulose from cornstalk, beet root and carrot waste.

Regarding the formulation of biopolymers indicated in Table 1, Figure 3 shows images of the obtained films.

Films containing starch showed a transparent appearance (B, C, D, E), whereas films containing cellulose (F, G, H, I, J, K, L, M, N, Ñ, O, P) were opaque, increasing opaqueness with increasing cellulose content. In turn, films with starch and cellulose (Q, R, S, T) showed a predominance of starch; however, films containing cellulose from carrot and beet showed opacity, due to cellulose presence.

### 3.2. Physicochemical Properties of Bioplastic Films

#### 3.2.1. Surface Morphology

**Figure 4:**
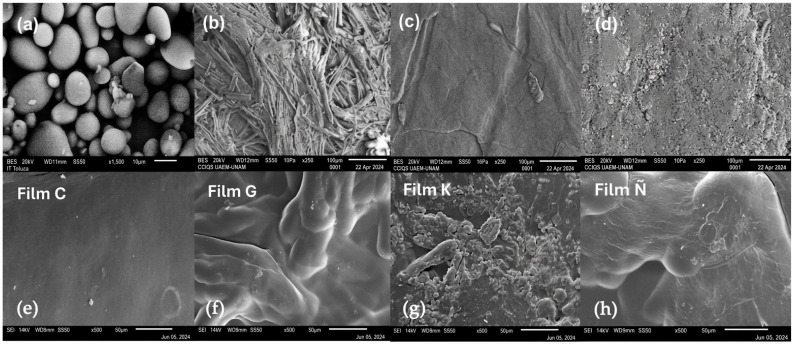
SEM micrographs of the raw materials and synthesized films according to Table 1 at different magnifications. (**a**) Starch from avocado seeds (×1500), (**b**) cellulose from cornstalk (×250), (**c**) cellulose from beet (×250), (**d**) cellulose from carrot (×250), (**e**) film C, (**f**) film G, (**g**) film K, (**h**) film Ñ at 500× of magnification.

Figure 4 shows SEM micrographs of the raw materials corresponding to (a) starch from avocado seed, (b) cellulose from cornstalk, (c) cellulose from beet, and (d) cellulose from carrot. Figure 4 also shows micrographs of selected synthesized films, including film C (containing starch), G (containing cellulose from cornstalk), K (containing cellulose from beet) and Ñ (containing cellulose from carrot). Films B, E, F, were like C; films H, J, K were similar to G; films J, L, M were like K, and films N, O, P were like Ñ, whereas films Q, R, S, T containing combined starch and cellulose from each waste did not show differences with films G, K and Ñ. 

The morphological appearance from starch was similar to Martins et al. [22], Aráujo et al. [23], and Dios-Avila et al. [40], exposing typical granules of elliptical form, free of pores.

Cellulose from cornstalk showed elongated fibers. The beet cellulose has an irregular and rough morphology [27]. Carrot cellulose shows a smoother and more uniform surface [12]. It is observed that, after the completion of the hydrolysis, pure cellulose was obtained [41].

The synthesized films exhibited a different surface morphology, which were dependent on the material formulations, including differences in [30]. Films C and G containing starch and cellulose from cornstalk showed a compact morphology and uniform surface, which may well describe high mechanical strength, high hydrophobicity and lower permeability.

In contrast, films K and Ñ, containing celluloses from beet and carrot, exhibit opposite morphological characteristics, showing weak assembly with a non-uniform surface; thus, these films could present a frail mechanical resistance [30,42].

#### 3.2.2. Functional Groups Identification

**Figure 5:**
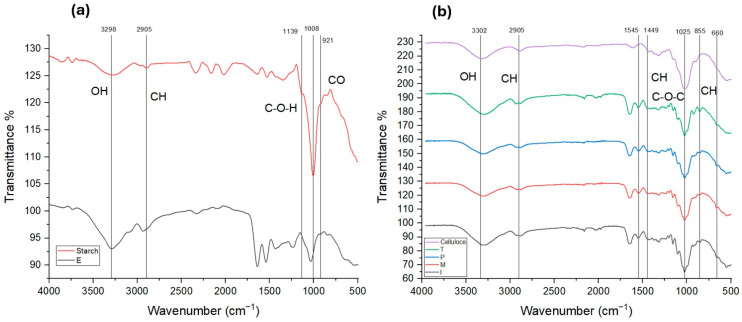
FTIR spectra of films (**a**) starch and film E. (**b**) Cellulose and films I, M, P, T.

Figure 5 shows the FTIR spectra with the main bands of chemical groups correspondent to starch and cellulose, as well as film E (containing starch), films I, M, P (containing cellulose from cornstalk, beet, carrot) and film T (containing starch and cellulose).

The FTIR spectrum of starch shows pronounced peaks of OH groups in 3692 cm^−1^ [43], while C-O bonds were observed around 921 cm^−1^, whereas C-O-C bonds were observed in 1025 cm^−1^ [3,23,44,45]. C-H bonds, showing the presence of glucose ring vibrations, were also detected in 855 cm^−1^ [3,23].

The detected groups of cellulose were OH, CH and CO. OH groups from cellulose are observed in 3300 cm^−1^, while CH is found in the three ranges 1500–1400 cm^−1^, 1100–1000 cm^−1^ and 900–800 cm^−1^, and CO is seen in 1024 cm^−1^ [11,27,30].

The spectrum of film E showed similar groups to those of starch, corroborating its presence, whereas films I, M, and P showed spectra like cellulose. However, all film spectra showed high peaks of OH and CH-O groups, describing predominantly physical interactions of OH from glycerol–gelatin–starch, glycerol–gelatin–cellulose and interactions between glycerol–gelatin–starch–cellulose, indicating intermolecular forces, most likely from hydrogen bonds with different chemical groups.

#### 3.2.3. Hydrophobicity

**Figure 6:**
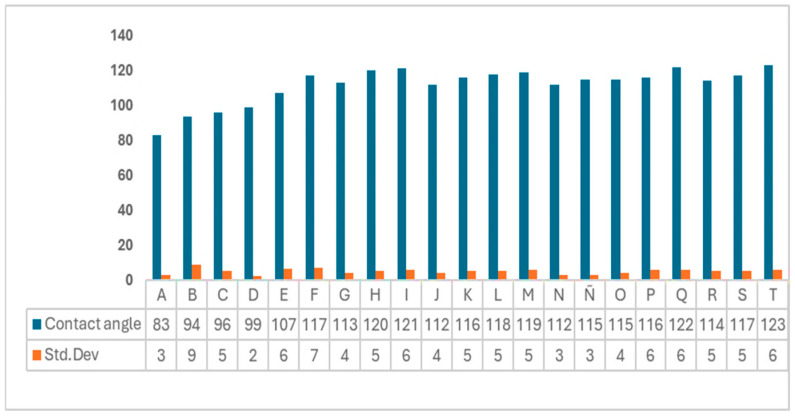
Contact angle of bioplastic films, including standard deviation.

Figure 6 shows the water contact angles of the bioplastic films. Unlike film A (control), which exhibited hydrophilic properties (83°), the films obtained with starch from avocado seed and cellulose from cornstalk, beet and carrot showed more hydrophobic properties, which is essential for the intended application, since the biggest obstacle for biopolymers is water absorption and rapid solubility in aqueous environments.

According to the data, film hydrophobicity was in the following order: T > Q > I > H > M > L, with an exposure range of water contact angle of 110–123°.

The hydrophobicity of film T was associated with composition, content, and strong interactions of the functional groups gelatin–starch–cellulose. In turn, film Q could exhibit high hydrophobicity, due to gelatin–cellulose interaction; however, the possible residual lignin or waxes in cornstalk cellulose also cause C-C and C-O-C interactions from aromatic subunits, resulting in a hydrophobic biopolymer.

Films I and H, containing cellulose from cornstalk, exhibit lower hydrophobicity, followed by films M and L, which contain cellulose from beet, whereas films with starch and cellulose from carrot showed the lowest hydrophobicity.

The previous study by Sangsawang et al. [46] showed that water contact angles depend on the composition of materials. In this case, vanadium metallopolymer films (made by starch, chitosan and soaked in vanadium salt) exhibited hydrophobic properties, resulting in water contact angles up to 103°.

In turn, Mukaila et al. [47] found that the water contact angle of films is impacted by PLA/starch/lecithin interactions. Also, Mroczkowska et al. [8] showed that potato starch in films of gelatin and peel from the tomato waste enhanced hydrophobicity (greater than 90°), whereas Olarte-Paredes et al. [48] obtained null hydrophobicity in films made from (PVA/chitosan) reinforced with conductive fillers (55–69°).

#### 3.2.4. Water Permeability

Table 2 exhibits data of film water permeability % and swelling ratio %. In general, films B–T were less permeable than film A, which showed high water absorption, 50% permeability and 82% swelling ratio, due to gelatin presence. Films B–T showed ranges of permeability of 16–33% and a swelling ratio of 35–65%.

The lowest permeability % and swelling ratio % was observed in films T < I < H < Q < G, which corresponds to films elaborated with starch and cellulose from cornstalk. The obtained data confirmed the correlation between contact angle and structure, indicating that these films have a reduced absorption capacity (19–24%) due to the presence of cellulose from cornstalk.

In contrast, films with only starch (B–E) and cellulose from carrot waste (N–P) exhibited more absorption capacity (28–33%), whereas other films showed permeability averaging 24–28% and a swelling ratio of 42–62%.

Other reported biofilms have shown similar range values of swelling ratio and water solubility; however, they were elaborated with other formulations and raw materials. For example, Mirpoor et al. [49] obtained bioplastics from protein extracts of seed oil by-products, showing 41–53% swelling, while Ranote et al. [50] reported ranges of 55.63% of permeability and a minimum solubility of 25.67% of films from Moringa oleifera gum/poly (vinyl alcohol), which were dependent on the components’ concentration. Mroczkowska et al. [8] showed swelling ratios between 48 and 58% from films of cutin-coated bioplastics, achieving a water solubility of 60 % after 24 h. Additionally, Pérez-Marroquín et al. [51] prepared films based on starch and gelatin, showing 30% of water solubility, which was similar to film B containing gelatin and starch from avocado seeds.

#### 3.2.5. Mechanical Properties

**Figure 7:**
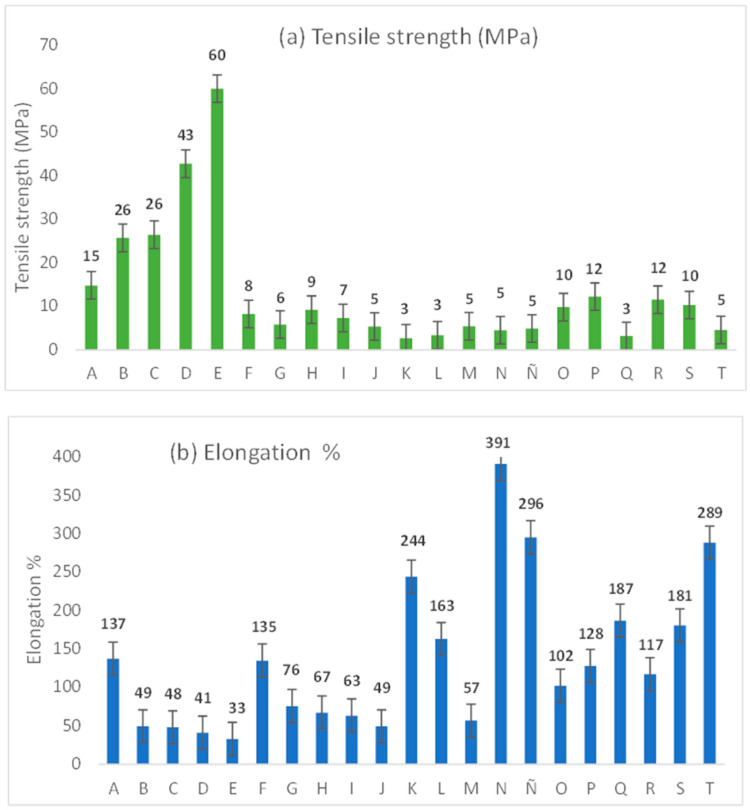
Mechanical properties of films, including tensile strength (MPa) and elongation %.

Figure 7 shows the mechanical properties of films, including tensile strength (MPa) and elongation (%). The highest tensile strength was achieved by film E (containing starch), followed by films F–I and R–S, showing less ductile properties and the lowest elongation capacity with the highest Young modulus. The mechanical behavior of these films was attributed to the formation of hydrogen bonds between amylose molecules from starch, and interactions between gelatin–glycerol–cellulose from cornstalk, providing a rigid assembly.

Other results showed that film K, L (containing cellulose from beet), N, Ñ (containing cellulose from carrot) and T (containing starch and celluloses) were highly deformable, indicating the highest ductile structure with the lowest Young modulus, as shown in Table 3.

According to the ASTM D882 standard, the films had thickness <1 mm; therefore, this parameter did not influence the results of the mechanical tests [52].

To complete the data, Table 3 and Table 4 show Young modulus and information from the ANOVA analysis and the comparison of the elongation response of films A–T using the Tukey test (*p* ≤ 0.05).

Films with starch (B–E) showed the lowest ductility, high rigidity and high Young modulus; however, the starch concentration between starch films (0.5–2 g) did not significantly affect the mechanical properties.

Films with cornstalk cellulose (F–I) also showed a reduction in ductility, indicating a change in mechanical resistance. In this case, a significant effect of cellulose content was observed between films F and I.

In turn, films with beetroot cellulose (J–M) exhibited elongation properties; films L and M, especially, showed significant changes, due to the cellulose concentration.

Regarding the films with carrot cellulose, films N and Ñ have the highest elongation percentage in comparison with the other films, including film A. In this case, cellulose concentration caused significative differences between films.

Between films Q and T, Q displayed lower percentage elongation, due to the presence of starch and cellulose from cornstalk, whereas film T presented a high percentage elongation, because it has celluloses from beet and carrot.

According to the data, the tensile strength of films B–E (26–60 MPa) and R–S (10–12 MPa) could be comparable with conventional plastics, such as polyethylene (PE) (16–45 MPa), high-density polyethylene (HDPE) (9–54 MPa) and polypropylene (PP) (28–38 MPa).

In comparison with other reports, dissimilar mechanical properties have been obtained in preceding studies on biofilms, due to different composition. Gomes de Menezes et al. [53] reported a film of chitosan and cassava starch with tensile strength (39–50 MPa), which was comparable to films B–E.

Information on elongation from bioplastics is found in [54]. The authors elaborated a bioplastic based in polyvinyl alcohol/graphene oxide, resulting in 268.64% of elongation, whereas Harunsyah et al. [55] reported 236.80% elongation in a film of cassava starch-based bioplastics reinforced with zinc oxide. Yahia et al. [56] reported 148% elongation in a bioplastic based on starch, polyvinyl alcohol, sorbitol, and cardan oil. Lubis et al. [57] developed a material using avocado seed starch reinforced with oil palm cellulose and glycerol, achieving an elongation at break of 13.36%; however, films B–T achieved elongation ranges 32–391%.

#### 3.2.6. Thermal Analysis

Table 5 shows the thermal behavior by TGA and DSC analysis of selected films A, E, I, M, P, and T. Other films did not show significant changes in the thermal properties with respect to film A.

TGA analysis involves degradation temperatures (DTs) with changes in mass, which are indicated as the first change in mass due to water loss (DT1), the second change due to mass loss of material (DT2) and the third change due to total mass loss and carbonaceous residue (DT3).

In addition, DSC analysis includes glass transition temperature (GTT), crystallization temperature (CT) and melting temperature (MT).

Film A showed the lowest degradation temperatures; however, films E–T increased these parameters. The DT1 range of films E–T was 83.9–114.5 °C.

Specifically, films I, M, and P, containing cellulose from cornstalk, beet, and carrot, respectively, showed the highest DT1, indicating that these films require elevated temperature for water evaporation. Due to their structure, they retain more water. However, films E and T, containing starch, showed lower DT1 values than films I, M, and P.

In turn, the DT2 range for films E–T was 139–217 °C, where film P containing cellulose from carrot offered higher resistance to mass loss. In contrast, films E and T with starch and cellulose showed the lowest DT2, meaning faster gas formation and mass loss. This result was attributed to the presence of starch in film E and the weakness of film T, due to multicomponent starch and cellulose.

The DT3 for films occurred in the range of 270–345.9 °C. Films I and M with cellulose from cornstalk and beet, respectively, showed the highest temperature for total mass loss. Therefore, they have superior resistance to pyrolysis. On the other hand, films P and T with cellulose from carrot and starch and cellulose, respectively, presented the lowest DT3, because cellulose from carrot provided a weaker structure; a similar behavior was obtained with starch and cellulose.

Regarding thermal behavior by DSC analysis, films E–T registered different data of GTT, CT and MT.

GTT in films E–T increased in comparison with film A, as follows: E > I > M > P > T, indicating that these films change from a glassy state to a rubbery state at more elevated temperatures than film A. However, GTT in film T containing starch and celluloses showed a similar value to film A, due to the integration of starch or cellulose or both.

On the other hand, CT values in films E–T decreased in comparison with film A, indicating that the integration of starch and cellulose reduced the temperature from a liquid or amorphous state to a crystalline state. The lowest CT was for film P containing cellulose from carrot.

In turn, the MT was also decreased in comparison with film A. In this case, film P registered the lowest MT, showing that, at 273 °C, the material changes from a solid to a liquid state.

**Figure 8:**
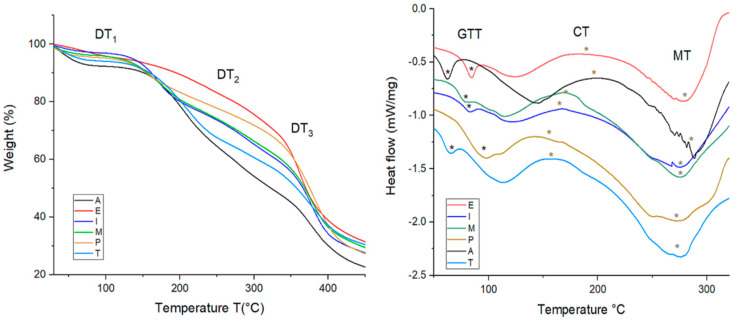
TGA and DSC thermograms of featured films, showing changes in the behavior with the temperature. A (control), E (starch from avocado seeds), I (cellulose from cornstalk), M (cellulose from beet root waste), P (cellulose from carrot waste) and T (combined starch from avocado seeds and cellulose from cornstalk).

Figure 8 includes thermograms of TGA and DSC of films A, E, I, M, P, and T.

The TGA values of the starch film (E) were comparable with the reported data from films of potato starch (Lopes et al. [7]) indicating variable ranges of DT1 as 80.4–185.7 °C, DT2 as 97.1–251.5 °C, and DT3 as 300 °C.

In turn, film M (containing cellulose from beet) was like those found by Yun et al. [58]. The authors reported citrus peel powder-based films with sodium alginate, indicating the first stage of decomposition DT1 at 100 °C, whereas DT2 was 203 °C; however, DT3 showed the highest value, which was associated with the highest content of carbonaceous waste and lignin.

Sequentially, film P containing cellulose from carrot was comparable with the results reported by Morán et al. [41]; however, they reported data from nanocellulose extracted from sisal fibers, indicating DT1 at 150 °C, DT2 at 200 °C and DT3 occurring at 305 °C.

Furthermore, the thermal stability from films with cellulose from cornstalk was like films made of corn starch and licorice root, observing a GT of 81 °C [59]. However, thermal stability from film E was not comparable with the data reported by Liu et al. [60]. The authors reported the lowest CT/°C (100 °C) and MT/°C (110 °C) for potato starch film, which was attributed to starch source.

### 3.3. Film Application in Cheese Storage

**Figure 9:**
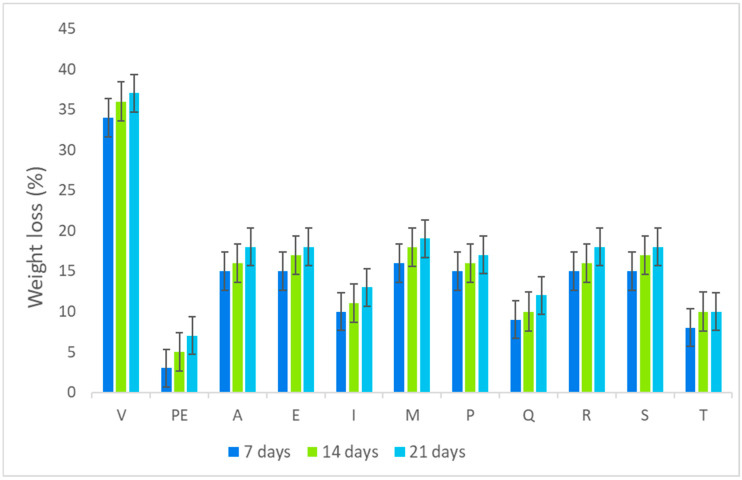
Kinetics of percentage weight loss of cheese after 7, 14, and 21 days of storage with films A–T as wrapping and PE and V (controls).

Figure 9 shows the weight loss behavior of Manchego cheese stored wrapped in films A–T, including PE (wrapper control, as conventional plastic) and the cheese sample control (V) without wrapping, during 21 days of storing at 4 °C.

As expected, after 6 days of storage, the cheese sample control without wrapping (V) showed the highest dehydration with the highest weight loss (30–35%). On the other hand, the control film PE maintained freshness and cheese quality with the lowest weight loss (4%), exhibiting a high permeation barrier and moisture resistance to prevent the cheese from losing weight and drying out.

In turn, films T, Q, and I (containing starch and cellulose from cornstalk) exhibited the best protection of cheese among the synthesized films, showing ranges of 8–15% of weight loss.

The other cheese samples wrapped with films A, E, M, P, R, and S (containing only starch and cellulose from beet and carrot) showed higher ranges of weight loss (15–18%). In addition, the wrapped cheese samples with these films showed yellowing behavior on the edges, showing low barrier permeation.

In general, the response of films A–T on cheese protection was associated with the use of glycerol (plasticizers), preventing the formation of pores and cracks, and uniform structure. However, films T, Q, and I showed the formation of a high barrier protection, due to high interactions of the glycerol–starch–cellulose from cornstalk, rendering bioplastic films with high water barrier properties.

Based on these results, films T, Q, and I could be used as wrapping material for Manchego cheese, since they meet the requirements of breathability, control humidity, and prevention of water condensation. However, more studies are necessary to enhance the formulation of films and to evaluate other cheese properties. In addition, they could also be tested as wrapping for other foods.

Previous studies of Lopes et al. [7] showed that elaborated biofilms with carob and potato waste protected the cheese samples during their storage; however, after 10 days, they showed partial conservation. Jridi et al. [29] also assessed the behavior of a biofilm based in gelatin–pectin as cheese wrapping; in this case, the authors found that the film allowed air circulation in the sample, causing cheese dehydration.

### 3.4. Film Degradability

Table 6 shows the degradation % of the films A–T over time by biological activity (B) and physicochemical factors of water and sun (W/S), water and air (W/A) and sun and air (S/A). The data corresponds to the average of three film samples with a standard deviation of ±1–5 days.

The most biodegradable material was film A; however, films E–T were also highly biodegradable, concluding their degradation after 20–40 days, because they contain starch and cellulose, retarding their biological degradation [61] in comparison to film A.

Regarding film degradation by physicochemical factors, films E, P, and T were very degradable by combined factors W/A and W/S, achieving 100% of degradation in 30 days. However, the degradation of films I and T by S/A was completed in 38 days, due to the presence of cellulose from cornstalk.

According to degradability information, films A–T were highly degradable; therefore, they can be used as food wrapping as an environmentally friendly option.

Different studies have reported the degradability of biopolymer materials, showing similar information to this investigation. Mohammed et al. [62] reported the degradation of alginate composite films in 14 days. Ibrahim et al. [63] indicated that films of corn starch and corn leaf fiber were biodegradable in 14 days. Jayalath et al. [64] found that composites of gelatin and cassava starch degraded 67% in 7 days, while the corn–rice starch films degraded 51.81% at the same time, whereas corn composite degradation was 47.72% after 14 days, showing the highest degradability resistance.

## 4. Conclusions

This study demonstrated the successful synthesis of biodegradable bioplastic films derived from agro-industrial waste, including avocado seed starch and cellulose extracted from cornstalk, carrot, and beet waste, contributing to waste valorization and circular economy strategies. The recovery yields of starch (25%) and cellulose (24–52%) confirmed the technical feasibility of transforming food-processing waste into sustainable polymeric materials for packaging applications.

Film properties were strongly influenced by the interactions among starch, cellulose, gelatin, and glycerol within the polymeric matrix. Film E, formulated with a higher avocado seed starch content, exhibited the highest tensile strength (60.12 ± 4.53 MPa) and Young’s modulus (355.26 ± 12.98 MPa). This enhanced mechanical performance is attributed to the reinforcing effect of starch, whose abundant hydroxyl groups promoted dense hydrogen bonding with gelatin and plasticizer molecules. These interactions reduced polymer chain mobility, generating a compact and rigid structure capable of resisting mechanical stress.

Conversely, film N, containing the lowest filler content (0.5 g cellulose), showed the highest elongation at break percentage (390.82 ± 14%), indicating superior flexibility. The reduced reinforcement allowed greater molecular mobility and efficient stress dissipation during deformation, resulting in improved ductility.

Regarding surface behavior, film T showed the highest water contact angle (123°), and thus the highest hydrophobicity. This effect was associated with strong starch–cellulose interactions and the presence of residual lignocellulosic components, which decreased surface polarity and limited water affinity.

Furthermore, selected films effectively preserved cheese moisture and achieved complete biodegradation within 20–40 days, highlighting their potential as eco-friendly alternatives to conventional plastic packaging.

Overall, the results demonstrate that avocado seed starch mainly contributes to mechanical strength, whereas carrot cellulose improves film flexibility, and cornstalk cellulose enhances hydrophobicity, allowing the development of bioplastics with tunable properties depending on formulation. To the best of our knowledge, there are currently no reported studies combining these agro-industrial residues for the development of biodegradable films for food packaging applications.

## Figures and Tables

**Table 1:** Film formulations, using water, glycerol and gelatin as base material (film control A) and incorporation of avocado seed starch and cellulose from carrot, beet and cornstalk.

Film Number		1	2	3	4	5	6	7	8	9	10	11	12	13	14	15	16	17	18	19	20	21
Film identification		A	B	C	D	E	F	G	H	I	J	K	L	M	N	Ñ	O	P	Q	R	S	T
Starch content (g)		0	0.5	1	1.5	2	0	0	0	0	0	0	0	0	0	0	0	0	0.5	0.5	0.5	0.25
Cellulose content	Cornstalk	0	0	0	0	0	0.5	1	1.5	2	0	0	0	0	0	0	0	0	0.5	0	0	0.25
	Beet	0	0	0	0	0	0	0	0	0	0.5	1	1.5	2	0	0	0	0	0	0.5	0	0.25
	Carrot	0	0	0	0	0	0	0	0	0	0	0	0	0	0.5	1	1.5	2	0	0	0.5	0.25

**Table 2:** Water permeability of films.

Films	A	B	C	D	E	F	G	H	I	J	K	L	M	N	Ñ	O	P	Q	R	S	T
Permeability (%)	50	33	33	28	28	25	24	22	20	31	30	30	28	30	30	28	27	23	25	25	19
Std.Dev.	0.25	0.20	0.24	0.22	0.21	0.19	0.21	0.11	0.11	0.23	0.22	0.24	0.15	0.17	0.11	0.09	0.11	0.19	0.14	0.16	0.15
Swelling ratio (%)	82	65	65	58	58	55	48	45	43	60	57	55	50	55	55	52	52	46	48	48	40
Std.Dev.	0.61	0.56	0.53	0.51	0.50	0.53	0.48	0.48	0.43	0.51	0.47	0.43	0.46	0.49	0.48	0.38	0.39	0.46	0.47	0.44	0.46

**Table 3:** Analysis of mechanical properties.

Sample	Strength (MPa)	Elongation (%)	Young’s Modulus	Thickness
			MPa	Mean (mm)
A	14.85 ± 3.23	137.16 ± 8	41.342 ± 18.19	0.032 ± 0.002
B	25.76 ± 3.06	49.28 ± 4	131.334 ± 23.36	0.044 ± 0.004
C	26.49 ± 4.24	48.13 ± 4	156.550 ± 25.80	0.052 ± 0.006
D	42.8 ± 2.34	41.03 ± 3	244.104 ± 24.55	0.055 ± 0.004
E	60.12 ± 4.53	32.89 ± 2	355.259 ± 12.98	0.064 ± 0.005
F	8.25 ± 1.65	134.66 ± 7	23.475 ± 5.10	0.078 ± 0.018
G	5.84 ± 1.87	75.56 ± 8	27.91 ± 9.32	0.164 ± 0.102
H	9.24 ± 1.17	67.27 ± 6	45.215 ± 4.07	0.130 ± 0.032
I	7.33 ± 0.79	63.45 ± 5	38.468 ± 5.54	0.027 ± 0.027
J	5.36 ± 1.38	49.28 ± 3	20.514 ± 4.07	0.105 ± 0.023
K	2.67 ± 0.33	244.05 ± 11	3.279 ± 1.24	0.132 ± 0.023
L	3.35 ± 1.44	162.71 ± 9	7.812 ± 4.53	0.143 ± 0.021
M	5.42 ± 2.12	56.74 ± 5	28.928 ± 0.18	0.177 ± 0.089
N	4.51 ± 0.97	390.82 ± 14	2.611 ± 1.11	0.082 ± 0.008
Ñ	4.90 ± 0.69	295.54 ± 12	4.768 ± 1.18	0.097 ± 0.008
O	9.82 ± 1.73	101.93 ± 7	32.933 ± 12.43	0.078 ± 0.013
P	12.25 ± 3.44	127.58 ± 7	65.09 ± 21.39	0.090 ± 0.024
Q	3.17 ± 0.73	186.79 ± 10	5.62 ± 1.89	0.093 ± 0.024
R	11.55 ± 1.89	116.81 ± 7	38.687 ± 12.69	0.090 ± 0.013
S	10.31 ± 1.07	180.59 ± 9	24.278 ± 11.58	0.070 ± 0.013
T	4.58 ± 0.55	288.60 ± 12	4.82 ± 1.38	0.098 ± 0.008

**Table 4:** Tukey comparation of deformation (%) in films (*p* ≤ 0.05).

Starch Films	Significance	Cornstalk Cellulose Films	Significance	Root Beet Cellulose Films	Significance	Carrot Cellulose Films	Significance	Starch and Cellulose Films	Significance
A–B	Significant	A–F	Significant	A–J	Non-significant	A–N	Significant	E–I	Non-significant
A–C	Significant	A–G	Significant	A–K	Significant	A–Ñ	Significant	E–M	Non-significant
A–D	Significant	A–H	Significant	A–L	Significant	A–O	Significant	E–P	Non-significant
A–E	Significant	A–I	Significant	A–M	Significant	A–P	Significant	E–T	Significant
B–E	Non-significant	F–G	Non-significant	J–K	Non-significant	N–Ñ	Significant	I–M	Non-significant
B–D	Non-significant	F–H	Significant	J–L	Significant	N–O	Significant	I–P	Non-significant
B–E	Non-significant	F–I	Significant	J–M	Non-significant	N–P	Significant	I–T	Significant
C–D	Non-significant	G–H	Significant	K–L	Significant	Ñ–O	Significant	M–P	Non-significant
C–E	Non-significant	G–I	Non-significant	K–M	Significant	Ñ–P	Significant	M–T	Significant
		H–I	Non-significant	L–M	Non-significant	O–P	Non-significant	P–T	Significant

**Table 5:** Thermal behavior of films by TGA and DCS analysis.

Films	DT_1_/°C	DT_2_/°C	DT_3_/°C	GTT/°C	CT/°C	MT/°C
A	70.9 ± 0.5	149.1 ± 0.1	251.3 ± 0.1	61.5 ± 0.2	197.0 ± 0.1	285.0 ± 0.1
E	95.6 ± 0.3	154.9 ± 0.5	332.9 ± 0.5	84.7 ± 0.3	183.1 ± 0.1	278.0 ± 0.1
I	114.5 ± 0.2	192.2 ± 0.1	345.9 ± 0.5	82.9 ± 0.5	166.0 ± 0.1	276.6 ± 0.3
M	101.4 ± 0.2	201.3 ± 0.1	340.7 ± 0.3	78.1 ± 0.1	168.4 ± 0.2	274.1 ± 0.1
P	100.2 ± 0.1	217.5 ± 0.2	313.3 ± 0.1	71.3 ± 0.1	142.8 ± 0.4	250.0 ± 0.1
T	83.9 ± 0.4	139.0 ± 0.1	270.0 ± 0.1	64.6 ± 0.3	155.6 ± 0.3	273.0 ± 0.1

**Table 6:** Degradation % of films over time by biological (B) factors and the effect of, simultaneously, physicochemical factors, water and solar light (W/S), water and air (W/A), solar light and air (S/A).

Days of Exposure	10				20				30				40			
Film/Degradation	B	W/S	W/A	S/A	B	W/S	W/A	S/A	B	W/S	W/A	S/A	B	W/A	W/S	S/A
A	100	50	70	30	100	100	100	50	100	100	100	60	100	100	100	100
E	80	35	30	20	100	50	60	50	100	70	80	70	100	100	100	90
I	60	25	20	10	80	50	30	35	100	80	70	60	100	100	100	83
M	70	50	60	50	90	70	80	70	100	100	100	100	100	100	100	100
P	80	60	70	20	100	90	90	40	100	100	100	70	100	100	100	90
T	65	50	60	20	80	80	80	35	100	100	100	55	100	100	100	85

## Data Availability

The original contributions presented in this study are included in the article. Further inquiries can be directed to the corresponding author.

## Abbreviations

The following abbreviations are used in this manuscript:

PE	Polyethylene
V	Unwrapped
B	Biological activity
W/S	Physicochemical factors of water and sun
WA	Physicochemical factors of water and air
S/A	Physicochemical factors of sun and air
DTs	Degradation temperatures
DT1	First change in mass by water loss
DT2	Second change in mass by water loss
DT3	Third change by total mass loss
GTT	Glass transition temperature
CT	Crystallization temperature
MT	Melting temperature
Wi	Initial weight
Wf	Final weight
